# Intranasal Esketamine Versus Other Pharmacological Strategies in Treatment-Resistant Depression with High Suicide Risk: A Six-Month Naturalistic Study

**DOI:** 10.3390/clinpract16060110

**Published:** 2026-06-12

**Authors:** Ana María de Granda-Beltrán, Alejandro Porras-Segovia, Daniel Núñez-Arias, Alba Rodríguez-Jover, Maria Paula Jassir Acosta, Philippe Courtet, Enrique Baca-García, Inmaculada Peñuelas-Calvo

**Affiliations:** 1 Department of Psychiatry, Hospital Universitario Fundación Jiménez Díaz, 28040 Madrid, Spain; anagrandabltran@gmail.com (A.M.d.G.-B.); arodriguez@fjd.es (A.R.-J.); research.fjd@gmail.com (E.B.-G.); 2 Mental Health Research Group, Health Research Institute Jimenez Díaz Foundation, 28040 Madrid, Spain; 3 Department of Psychiatry, Hospital Universitario Rey Juan Carlos, 28933 Móstoles, Madrid, Spain; 4 Institut National de la Santé et la Recherche Medicale (INSERM), 34000 Montpellier, France; p-courtet@chu-montpellier.fr; 5 Department of Neuroscience and Health Sciences, Universidad Carlos III de Madrid (UC3M), 28903 Madrid, Spain; 6 Department of Psychiatry, Hospital Universitario de Ferrol, 15405 Galicia, Spain; dnunezariascoruna@gmail.com; 7 Department of Child and Adolescent Psychiatry, Hospital Universitario 12 de Octubre, 28041 Madrid, Spain; mariapaula.jassir@salud.madrid.org (M.P.J.A.); inmaculada.penuelas@salud.madrid.org (I.P.-C.); 8 Department of Psychiatry, Hospital Universitario Infanta Elena, 28342 Valdemoro, Madrid, Spain; 9 Department of Psychiatry, Hospital General de Villalba, 28400 Villalba, Madrid, Spain; 10 CIBERSAM-ISCIII (Biomedical Research Networking Centre for Mental Health), 28029 Madrid, Spain; 11 Department of Psychiatry, Universidad Autónoma de Madrid, 28049 Madrid, Spain; 12 Health Research Institute Hospital 12 de Octubre (i+12 Institute), 28041 Madrid, Spain; 13 Department of Psychiatry, Universidad Complutense de Madrid, 28040 Madrid, Spain

**Keywords:** esketamine, treatment-resistant depression, suicidal behavior, naturalistic study

## Abstract

**Background:** Treatment-resistant depression (TRD) poses a major clinical challenge, particularly when accompanied by suicidal behavior. Intranasal esketamine has demonstrated rapid antidepressant effects in TRD, but real-world comparative evidence remains limited. **Methods:** We conducted a six-month naturalistic prospective cohort study in two Spanish mental health centers, including 62 TRD patients with high suicide risk undergoing fourth-line treatment. Thirty patients received intranasal esketamine and thirty-two alternative pharmacological interventions. Suicidal ideation (C-SSRS), depressive symptoms (HAM-D-17) and functional status (FAST) were assessed at baseline and at 1-, 3- and 6-month follow-ups. **Results:** Both groups showed significant improvement during follow-up; however, esketamine-treated patients exhibited a faster and greater reduction in suicidal ideation and depressive symptoms than those receiving alternative pharmacological strategies. The number needed to treat to prevent one case of high suicide risk was 1.35. Functional improvement was comparable between groups. **Conclusions:** In real-world clinical settings, intranasal esketamine was associated with a faster and greater reduction in suicidal ideation and depressive symptoms among TRD patients with high suicide risk, supporting its role as a rapid-acting therapeutic option within comprehensive and closely monitored care.

## 1. Introduction

Major depressive disorder (MDD) is one of the leading causes of disability worldwide, representing a significant public health problem due to its high prevalence, recurrent nature and substantial functional impairment [1]. Within this clinical spectrum, treatment-resistant depression (TRD) poses a particularly complex challenge, given that approximately 20–30% of patients fail to respond adequately after at least two correctly prescribed antidepressant treatments [2]. This situation is associated with greater chronicity, increased use of healthcare resources, and a significantly elevated risk of suicidal behavior [3]. Specifically, patients with MDD are 12–20 times more likely to commit suicide than the general population [4] and response to treatment is worse when MDD is associated with suicidal ideation [5].

In this context, suicidal ideation in TDR constitutes a clinical emergency. Conventional antidepressant treatments typically take several weeks to achieve a significant therapeutic effect, limiting their usefulness in high-risk situations [6]. Even after multiple augmentation or combination strategies, a significant proportion of patients continue to experience severe depressive symptoms and persistent suicidal risk [2]. Thus, this pattern highlights the need for faster-acting interventions with alternative mechanisms of action.

In recent years, intranasal esketamine has emerged as an innovative therapeutic alternative for patients with TRD. Its mechanism of action involves antagonizing the NMDA receptor and modulating the glutamatergic system, resulting in rapid changes in synaptic neuroplasticity [7]. Randomised controlled clinical trials, such as the ASPIRE I and ASPIRE II studies, have shown a significant and rapid reduction in depressive symptoms and suicidal ideation in patients with MDD and acute suicidal risk when treated with intranasal esketamine alongside standard antidepressant treatment [8,9]. Similarly, the TRANSFORM study programme demonstrated the efficacy of intranasal esketamine compared to placebo when added to oral antidepressant treatment in patients with TRD [10]. Even though clinical trials provide robust evidence under controlled conditions, the generalisation of their results to routine clinical practice may be limited due to strict selection criteria and lower therapeutic heterogeneity [11].

In real-world clinical practice, patients with TRD often present with a greater burden of psychiatric and medical comorbidities, multiple prior treatment failures and greater overall clinical complexity than those typically included in randomised clinical trials [12]. Consequently, evidence from naturalistic studies evaluating the comparative effectiveness of intranasal esketamine versus other pharmacological strategies in routine care is limited. Therefore, the aim of this study was to compare the evolution of suicidal ideation and depressive symptoms in patients with TRD at high risk of suicide undergoing fourth-line treatment. Patients treated with intranasal esketamine were compared with those receiving alternative pharmacological strategies. Additionally, changes in overall functioning were explored. We hypothesised that treatment with intranasal esketamine would lead to a faster and greater reduction in suicidal ideation and depressive symptoms than conventional pharmacological strategies.

## 2. Materials and Methods

### 2.1. Context and Design

This is a naturalistic prospective observational comparative cohort study conducted in two outpatient psychiatric services in Spain: Jiménez Díaz University Hospital and the Ferrol University Hospital Complex. The study complied with the principles set out in the Declaration of Helsinki and was approved by the Research Ethics Committee (PIC065-24_HRJC, 12 June 2024) of Jiménez Díaz University Hospital. Written informed consent was obtained from all participating patients.

### 2.2. Sample

Our sample consisted of patients aged 18 years or older who met the European Medicines Agency (EMA) criteria for the diagnosis of TRD. The EMA defines TRD as a failure to achieve remission (rather than just a reduction in symptoms) after at least two separate adequate antidepressant trials (in different classes and at the proper dose and duration) during a single current episode of major depressive disorder [13]. In Spain, esketamine is only reimbursed as a fourth-line treatment, following the failure of two antidepressants and at least one augmentation or combination strategy [14]. All patients were considered to be at high risk of suicide and were enrolled, in most cases, in intensive follow-up programmes. Classification as being at high risk of suicide was based on a comprehensive clinical assessment by the treating psychiatrist, taking into account the severity of suicidal thoughts as measured by the C-SSRS, a history of previous suicide attempts, recent suicidal behavior or suicidal crises, and the need for intensive follow-up as part of a structured monitoring programme. These programmes involved frequent psychiatric visits and a comprehensive approach, incorporating both pharmacological and psychotherapeutic interventions. During the study period, 20 out of 30 patients (66.7%) in the esketamine group and 18 out of 32 patients (56.3%) in the other pharmacological strategies group received follow-up care from clinical psychology services. At the time of recruitment, all patients had previously received three treatment lines.

The inclusion criteria were as follows:

- 18 years of age or older.
- Meeting EMA diagnostic criteria for TRD.
- Initiation of a fourth-line pharmacological treatment after failure of three previous adequate treatment lines.
- Ability to understand the study procedures and provide written informed consent.
- Specific exclusion criteria:
- Presence of any condition that could interfere with participation in the study or the assessment of outcomes, according to clinical judgment.
- Inability to provide informed consent.

As this was a naturalistic observational study, treatment allocation was determined exclusively by the treating psychiatrist according to routine clinical practice and the individual needs of each patient. Consequently, some patients initiated intranasal esketamine, while others received treatment-as-usual pharmacological strategies, including antidepressant switching, combination therapies and augmentation approaches.

In the esketamine group, ongoing antidepressant treatment was maintained when esketamine was introduced. Prior to initiating intranasal esketamine or any fourth-line pharmacological strategy, patients had previously received a third-line treatment for an adequate duration and at therapeutic doses, with either non-response or only partial response observed, thereby justifying the need for a new treatment line. During the follow-up period, no new antidepressants or augmentation/combination pharmacological strategies were prescribed to patients receiving esketamine. In some cases, it was possible to reduce concomitant oral psychotropic medication due to clinical improvement.

### 2.3. Esketamine Treatment Procedure

Intranasal esketamine was initiated at a dose of 56 mg, in line with routine clinical practice and current prescribing recommendations. Dose escalation to 84 mg was considered after the first administration in patients who did not experience clinically significant adverse effects. Due to its generally good tolerability in our sample, most patients reached the 84 mg dose by the second or third administration. Only one patient remained on 56 mg throughout treatment because of more pronounced dissociative symptoms and dizziness following administration. Intranasal esketamine treatment sessions were administered once weekly throughout the six-month follow-up period under routine clinical supervision. All patients completed the six-month follow-up period as part of the study, and there were no discontinuations of treatment or dropouts due to adverse effects or lack of efficacy.

### 2.4. Measures and Outcomes

The independent variables included sociodemographic factors (gender, marital and employment status) and clinical factors (psychiatric diagnoses according to the 10th revision of the International Classification of Diseases (ICD-10), comorbidities and the number of previous suicide attempts).

The assessment instruments used were the Columbia–Suicide Severity Rating Scale (C-SSRS) to evaluate suicidal ideation; the 17-item Hamilton Depression Rating Scale (HAM-D-17) to assess the severity of depressive symptoms; and the Functioning Assessment Short Test (FAST) to evaluate functional status. These scales were administered at baseline (prior to treatment initiation) and at follow-up visits at one, three, and six months.

The primary outcomes were changes in suicidal ideation and depressive symptoms severity during the follow-up period in order to compare the effectiveness of intranasal esketamine with that of other pharmacological strategies in patients receiving fourth-line treatment; functional status was considered a secondary outcome.

### 2.5. Statistical Analysis

All statistical analyses were performed using SPSS statistical software (version 30.0). Descriptive statistics were calculated to characterise the study sample, and baseline characteristics were compared between the two groups. Changes in clinical scores on the Columbia–Suicide Severity Rating Scale (C-SSRS), the 17-item Hamilton Depression Rating Scale (HAM-D-17) and the Functioning Assessment Short Test (FAST) over the six-month follow-up period were analysed using a repeated-measures analysis of variance (ANOVA) to compare patients treated with intranasal esketamine with those receiving alternative pharmacological treatments. An independent-samples Student’s *t*-test was used to evaluate the difference in percentage clinical improvement and reduction in suicide risk between patients treated with intranasal esketamine and those receiving alternative therapeutic approaches. The number needed to treat (NNT) was also calculated. All statistical tests were two-tailed, with a significance level set at *p* < 0.05 and 95% confidence intervals.

## 3. Results

### 3.1. Baseline Characteristics of the Sample

A total of 62 patients were included in the study. Thirty received intranasal esketamine, while the remaining 32 were treated with alternative pharmacological strategies. All patients received standard clinical care for TDR, including at least one antidepressant (selective serotonin reuptake inhibitor (SSRI), serotonin–norepinephrine reuptake inhibitor (SNRI), or another type of antidepressant), augmentation or combination strategies, and psychotherapy when clinically indicated.

There were no statistically significant differences between the groups in terms of mean age (43.4 vs. 43.6 years; *p* = 0.974). However, the gender distribution differed significantly (*p* = 0.010): the esketamine group was predominantly male (70%), while the alternative-strategies group was predominantly female (62.5%).

No significant between-group differences were observed in employment status (*p* = 0.21). In the esketamine group, temporary disability leave was the most frequent situation (33.3%), followed by unemployment (26.7%). In the other strategies group, the most common categories were temporary disability leave (37.5%) and employment (25%). None of the patients in the esketamine group were permanently disabled, and none of the patients in the other-strategies group were retired.

Similarly, there were no significant differences in psychiatric comorbidities between groups (*p* = 0.18). In the esketamine group, the most common conditions were personality disorders (26.7%), dysthymia (13.3%), and autism spectrum disorder (13.3%). In the other strategies group, the most common conditions were personality disorders (28.1%), bipolar disorder (15.5%), and dysthymia or anxiety disorders (12.5% each).

The most frequently prescribed treatments in the other-strategies group were SNRIs (59.4%), followed by SSRIs (28.1%). Antipsychotic augmentation was used in 53.1% of patients and the most common pharmacological regimen was a combination of an SNRI and an antipsychotic (21.9%).

In the esketamine group, the most frequently used antidepressants for concomitant pharmacological treatment were SNRIs (50%), primarily venlafaxine (33.3%) and duloxetine (16.7%), followed by SSRIs (20%), for which fluoxetine was the most commonly prescribed agent (10%). Bupropion (10%), clomipramine (6.7%), vortioxetine (6.7%) and lithium (3.3%) were also used to a lesser extent. The main pharmacological combination strategy involved mirtazapine (23.3%), while 26.7% of patients received antipsychotic augmentation, primarily with aripiprazole (16.7%), quetiapine (6.7%) and lurasidone (3.3%). The most common combinations were venlafaxine plus mirtazapine (10%) and SNRIs combined with aripiprazole augmentation (13.3%).

The comparator group reflected routine fourth-line pharmacological management in treatment-resistant depression. The most frequently prescribed treatments were SNRIs (59.4%), specifically extended-release venlafaxine and, in two patients, duloxetine; as well as SSRIs (28.1%), with sertraline being the most commonly prescribed agent. Antipsychotic augmentation was used in 53.1% of patients and the most common regimen was a combination of an SNRI and an antipsychotic (21.9%). In our sample, aripiprazole was the most frequently used antipsychotic for augmentation. Other, less frequently employed strategies included vortioxetine, mirtazapine, lithium and tricyclic antidepressants, as well as various combination regimens.

Although the difference was not statistically significant (*p* = 0.068), the mean number of suicide attempts was higher in the other strategies group (2.35 ± 2.03) than in the esketamine group (1.26 ± 2.38). There were no significant differences in baseline C-SSRS scores (4.04 ± 1.15 vs. 3.56 ± 1.43; *p* = 0.102), FAST scores (48.7 ± 12.1 vs. 49.6 ± 8.9; *p* = 0.754), or HAM-D-17 scores (26.3 ± 5.27 vs. 28.3 ± 3.44; *p* = 0.089). Overall, the groups were comparable in most baseline sociodemographic and clinical characteristics, except for gender distribution. Baseline characteristics are presented in Table 1.

### 3.2. ANOVA Analysis of Repeated Measures Between Groups

A repeated measures ANOVA was conducted to evaluate how clinical scores changed over the six-month follow-up period for the two patient groups.

### 3.3. Suicidal Behavior

Regarding suicidal ideation, a statistically significant main effect of time was observed, indicating a reduction during the follow-up period in both groups (F = 57.06; *p* < 0.001; partial η^2^ = 0.55). The effect remained after adjusting for sex (F = 4.05; *p* = 0.09; partial η^2^ = 0.08) but disappeared after adding to the model age, number of suicide attempts, baseline depression severity, and baseline suicidal ideation intensity (*p* = 0.433).

Furthermore, patients treated with intranasal esketamine exhibited a more substantial overall reduction in suicidal ideation than those receiving alternative pharmacological treatments (F = 12.43; *p* < 0.001; partial η^2^ = 0.21). The effect remained after adjusting for sex, age, number of suicide attempts, baseline depression severity, and baseline suicidal ideation intensity (F = 21.00; *p* < 0.01; partial η^2^ = 0.37).

A statistically significant time × treatment interaction was also identified for the reduction in suicidal ideation (F = 11.78; *p* < 0.001; partial η^2^ = 0.20). This interaction remained statistically significant after adjusting for sex, age, number of suicide attempts, baseline depression severity, and baseline suicidal ideation intensity (F = 17.41; *p* < 0.01; partial η^2^ = 0.33).

**Figure 1:**
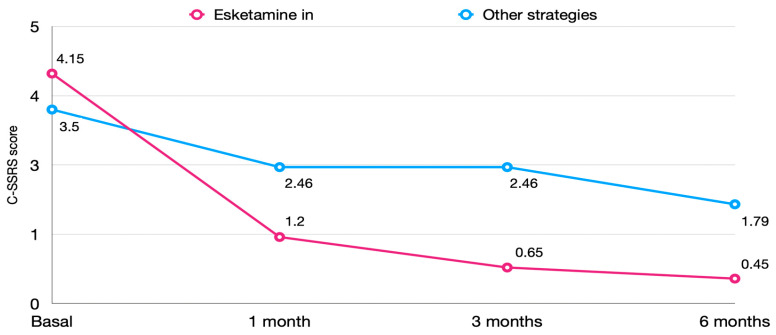
Evolution of the Columbia Suicide Severity Rating Scale score throughout the follow-up period.

After one month of treatment, mean suicide risk score decreased by 71% in the esketamine group, compared to 42.3% in the group receiving other strategies. Figure 1 illustrates the evolution of clinical scores comparing both groups.

### 3.4. Depressive Symptoms

A significant main effect of time was observed for depressive symptoms assessed with the HAM-D-17 scale, indicating a reduction in symptoms during follow-up in both groups (F = 46.71, *p* < 0.001, partial η^2^ = 0.50). The effect disappeared after adjusting for sex, age, number of suicide attempts, and baseline suicidal ideation intensity (*p* = 0.579).

**Figure 2:**
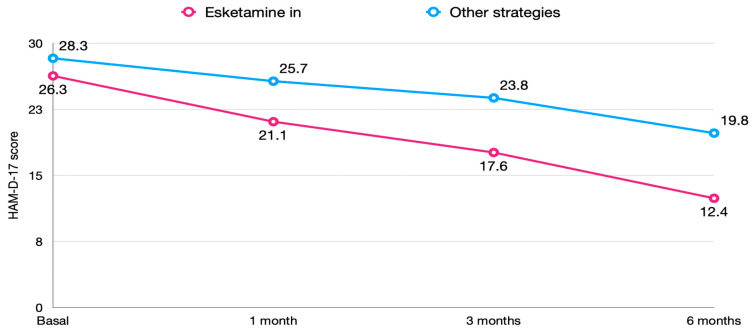
Evolution in Hamilton Depression Scale scores throughout follow-up.

Patients treated with esketamine exhibited significantly lower HAM-D-17 scores overall than those receiving alternative treatments (F = 18.78, *p* < 0.001, partial η^2^ = 0.29). The effect remained after adjusting for the above-mentioned variables (F = 13.24, *p* < 0.001, partial η^2^ = 0.26). There was also a significant time × treatment interaction (F = 3.10, *p* = 0.029, partial η^2^ = 0.06), which remained statistically significant after adjustment (F = 6.87, *p* = 0.013, partial η^2^ = 0.16. Figure 2 shows the trajectory of depressive symptoms over time comparing both groups.

### 3.5. Overall Functioning

A significant main effect of time was observed regarding overall functioning, as assessed using the FAST scale (F = 40.09, *p* < 0.001, η^2^ = 0.47). The effect disappeared after adjusting for cofounding factors (*p* = 0.786).

**Figure 3:**
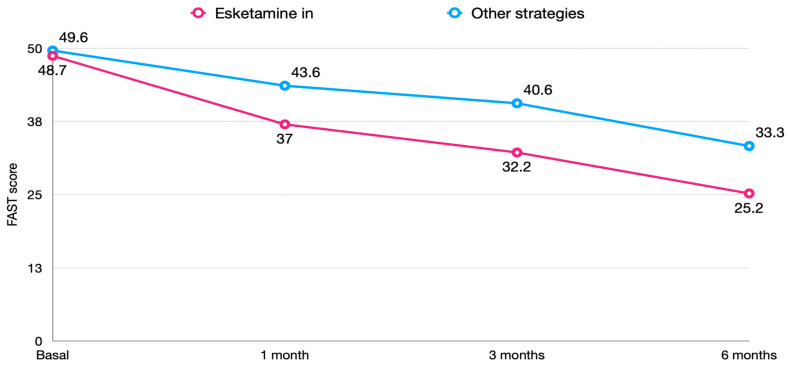
Evolution in the FAST functionality scale score throughout follow-up.

Patients treated with intranasal esketamine showed greater improvement in overall functioning (lower FAST scores) than those receiving other treatments (F = 4.43, *p* = 0.041, partial η^2^ = 0.09). The effect remained after adjusting for sex, age, number of suicide attempts, baseline depression severity, and baseline suicidal ideation intensity (F = 6.17, *p* = 0.018, η^2^ = 0.15). However, no significant time × group interaction was observed (F = 1.49, *p* = 0.219, partial η^2^ = 0.03). Figure 3 presents the changes in functional outcomes during follow-up comparing both groups.

### 3.6. Comparison Between Groups of Final Clinical Outcomes

Independent-samples Student’s *t*-tests were performed to evaluate differences in the percentage of clinical improvement and reduction in suicide risk between groups.

There was significantly greater clinical improvement in the esketamine group (88.6% ± 17.2% vs. 44.8% ± 52.5%; t(46) = 3.59; *p* < 0.001), with a large effect size (Cohen’s d = 1.05) [12]. Similarly, the reduction in suicide risk was significantly greater in the esketamine group (74.0 ± 26.0 vs. 34.5 ± 38.1; t(47) = 4.03; *p* < 0.001) and also had a large effect size (Cohen’s d = 1.17).

The number needed to treat (NNT) was calculated using the formula NNT = 1/Absolute Risk Reductio (ARR), where ARR = Control Event Rate (CER)—Experimental Event Rate (EER). An event was defined as persistent active suicidal ideation at the end of follow-up (a C-SSRS score of 2 or more). CER = 0.517. EER = 0.105. ARR = 0.412. The resulting NNT to prevent one case of high-risk suicidal ideation was 2.43 (95% CI: 1.56–5.44), indicating a potentially substantial clinical benefit of intranasal esketamine treatment.

### 3.7. Safety and Tolerability

Intranasal esketamine demonstrated a generally favorable tolerability profile in our study population. The most frequently observed adverse effects were mild dissociative symptoms and dizziness during the observation period after administration. Three patients experienced a mild, transient increase in blood pressure during this period, which resolved spontaneously without the need for further intervention. Adverse effects tended to decrease in intensity as treatment progressed. No patients experienced any clinically significant adverse effects after leaving the mental health center following treatment. Furthermore, no treatment discontinuations or dropouts related to adverse effects or tolerability issues were observed during the six-month follow-up period. Additionally, no emergency department visits or psychiatric hospitalizations were recorded during the esketamine treatment period.

## 4. Discussion

### 4.1. Summary of Results

In this study, we explored the evolution of suicidal ideation and depressive symptoms in patients with TRD who were treated with esketamine compared to those who received other pharmacological strategies in real-world clinical practice during a six-month follow-up period. We also evaluated the evolution of overall functioning throughout the follow-up period. Our findings revealed that patients with TRD and a high risk of suicide who received intranasal esketamine exhibited a notably quicker and more substantial reduction in suicidal ideation compared to those who received alternative pharmacological treatments.

Patients treated with esketamine also showed a significantly greater decrease in HAM-D-17 scale scores throughout follow-up. However, the two groups showed comparable functionality, suggesting that both treatment strategies were associated with similar improvements in overall functioning over time.

Regarding both suicidal ideation and clinical depression, our results suggest that a longer duration of esketamine treatment is associated with greater clinical improvement. However, this temporal pattern was not observed for functional outcomes, where the passage of time on treatment did not increase its effectiveness. It should also be noted that the NNT was low in the esketamine group for preventing cases of high suicide risk. This suggests a potentially substantial clinical benefit, although this finding should be interpreted with caution given the small sample size.

### 4.2. Comparison with Previous Evidence

Our findings are consistent with those reported in the scientific literature. The ASPIRE I and II clinical trials [8] were randomised, double-blind, placebo-controlled, multicentre studies that included patients aged 18–64 years with MDD and acute suicidal ideation or behavior. Patients were selected after presenting to the emergency department or an inpatient psychiatric unit. One group of participants received treatment with esketamine in conjunction with oral antidepressant treatment, and the other group received a placebo nasal spray in conjunction with oral treatment. Both groups showed a rapid reduction in suicide risk during the first month. In daily clinical practice, this effect has also begun to be demonstrated in relevant publications. Leonardi et al. [15] reported rapid and clinically significant reductions in suicidal ideation and depressive symptoms in 80 patients treated with intranasal esketamine in a real-world setting. Moreover, The REAL-ESKperience study [16] examined 236 outpatients treated with esketamine through a self-report survey, reporting early improvements in depressive symptoms and suicidal thoughts, with reduction in suicidal ideation being among the most prominent treatment effects.

Another noteworthy finding of our study was the presence of comorbidity in both treatment groups, as well as the persistence of the superior clinical response observed with intranasal esketamine, despite this clinical complexity. This is consistent with previous publications in the scientific literature, particularly the observational, retrospective, multicentre REAL-ESK study [17], which included 116 patients with TRD, 38% of whom had comorbidities. However, the study found no statistically significant differences in treatment response between patients with and without comorbidities.

Furthermore, our study observed that a longer duration of intranasal esketamine treatment was associated with greater reductions in both suicide risk and depressive symptoms. This finding is consistent with that previously reported by Conejero et al. [18], who observed that an increased number of esketamine administrations may enhance the likelihood of achieving clinical remission.

### 4.3. Potential Mechanisms for Our Findings

Unlike what occurred with suicidal ideation and depressive symptoms, functionality improved equally in both treatment groups. This could be explained by two factors. Firstly, the gradual improvements in daily functioning observed in both groups may be due to the improvements in depressive symptoms and reductions in suicidal ideation. Secondly, functional recovery often occurs more slowly than improvement in symptoms and may not be fully synchronised with the antidepressant response. Functional outcomes are frequently influenced by psychosocial and environmental factors, and improvements may continue even after symptomatic remission has been achieved [19,20]. The delayed and multifactorial nature of functional recovery could explain the absence of significant differences between treatment groups in functional outcomes.

### 4.4. Implications for Clinical Practice

These findings may have relevant implications for clinical practice. Suicide remains a major public health concern, and its therapeutic management is complex. The highest risk of suicide occurs in the first two months after a suicide attempt [21], highlighting the importance of rapid and effective therapeutic interventions during this critical period. In this regard, the data obtained suggest that intranasal esketamine might represent a rapid-acting therapeutic option for the management of suicide risk in patients with TRD, while improving depressive symptoms simultaneously.

### 4.5. Future Lines of Research

Recent studies have begun to explore the use of esketamine in adolescents, reporting promising preliminary results in the reduction in depressive symptoms and suicidal ideation [22,23]. Suicidal behavior is one of the most frequent reasons for psychiatric emergency consultations in children and adolescents, and there is also a high likelihood of recurrence in the first few months after treatment [24]. Therefore, given the need for rapid and effective strategies, further research into the use of esketamine in this population is necessary.

### 4.6. Limitations and Strengths of the Study

This study has several limitations. Firstly, the naturalistic, non-randomised observational design limits the ability to establish causal relationships and may introduce selection bias, as treatment allocation was based on clinicians’ judgement in routine care. Despite the absence of major baseline differences in illness severity between groups, residual confounding cannot be fully excluded. Secondly, the relatively small sample size might have reduced the statistical power to detect differences in certain secondary variables. In this regard, the observed NNT was exceptionally low, potentially reflecting the large effect size observed in a small sample. Therefore, this finding should be interpreted with caution and confirmed in studies with larger populations. Moreover, small samples and a high baseline risk profile can produce very low NNT values, which require confirmation in larger and more diverse cohorts. Thirdly, the significant difference in gender distribution between the groups is an additional potential source of confounding factors, given that sex-related differences in treatment response and suicidal behavior are well documented in the literature. Fourthly, while the proportion of patients receiving psychotherapeutic support was not formally compared between groups, the potential influence of psychotherapy on clinical outcomes cannot be ruled out. Fifthly, the comparator group consisted of heterogeneous treatment-as-usual pharmacological strategies rather than a standardised control intervention, which may have introduced confounding factors and reduced the internal validity of between-group comparisons. Sixthly, the absence of blinding represents another potential source of bias. Finally, the six-month follow-up period means that conclusions cannot be drawn about the long-term durability of the response to treatment, the risk of symptom relapse after treatment is stopped or the dose is reduced, or the long-term safety profile of intranasal esketamine compared with other fourth-line pharmacological strategies.

On the other hand, the study has several strengths. First, it provides real-world evidence regarding the effectiveness of intranasal esketamine in routine clinical practice, including patients with psychiatric comorbidities and complex clinical presentations, which enhances the external validity of the findings and reflects the heterogeneity typically observed in clinical settings. Thus, the study provides additional evidence of the effectiveness of intranasal esketamine in reducing depressive symptoms and decreasing suicidal ideation in patients with TRD in real-world clinical practice. Secondly, the sample consists of patients with TRD who are at high risk of suicide and receiving fourth-line treatment. This makes the findings clinically relevant, as the study focuses on a particularly severe and difficult-to-treat population for whom rapid-acting interventions are especially needed. Another notable strength is the use of validated scales during the longitudinal follow-up, which allows us to evaluate not only the magnitude of clinical improvement, but also the temporal trajectory of treatment response.

## 5. Conclusions

In this naturalistic cohort of patients with TRD and high suicide risk, treatment with intranasal esketamine was associated with clinically relevant improvements in depressive symptoms and suicidal behavior. Our findings suggest that, within the context of comprehensive care and intensive follow-up, esketamine may represent a rapid and effective fourth-line therapeutic option for this particularly severe population in routine clinical practice. However, given the observational design, limited sample size and follow-up duration, these results should be interpreted with caution and cannot be taken as definitive evidence of superiority over other fourth-line strategies. Further prospective, controlled, longer-term studies are needed to confirm these results and better understand the long-term effectiveness and safety of esketamine in patients with TRD at high risk of suicide.

## Figures and Tables

**Table 1:** Baseline characteristics of the sample.

	Esketamine (*n* = 30)	Other Strategies (*n* = 32)	*p*-Value Difference Between Groups
	**Mean (SD)**		
Age	43.4 (12.1)	43.6 (13.4)	0.974
No. of previous suicide attempts	1.26 (2.38)	2.35 (2.03)	0.068
Basal CSSRS	4.04 (1.15)	3.56 (1.43)	0.102
Basal FAST	48.7 (12.1)	49.6 (8.9)	0.754
Basal HAM-D-17	26.3 (5.27)	28.3 (3.44)	0.089
	*N* (%)		
Gender			0.010
Female	9 (30)	20 (62.5)	
Male	21 (70)	12 (37.5)	
Employment status			0.21
Actively employed	3 (10)	8 (25)	
Student	1 (3.3)	4 (12.5)	
Housewife	1 (3.3)	2 (6.3)	
TD	10 (33.3)	12 (37.5)	
Unemployed	8 (26.7)	3 (9.4)	
Disabled	0 (0)	2 (6.3)	
Retired	2 (6.7)	0 (0)	
Unemployed receiving unemployment benefits	1 (3.3)	1 (3.1)	
Comorbidity			0.18
No comorbidity	8 (26.7)	9 (28.1)	
PD	8 (26.7)	9 (28.1)	
Dysthymia	4 (13.3)	4 (12.5)	
Anxiety	1 (3.3)	4 (12.5)	
ASD	4 (13.3)	1 (3.1)	
ADHD	0 (0)	2 (6.3)	
BD	0 (0)	4 (15.5)	
Schizophrenia	1 (3.3)	0 (0)	
Other	3 (10)	3 (9.4)	
Other pharmacological strategies			
SSRIs	6 (20%)	9 (28.1)	
SNRIs	15 (50%)	19 (59.4)	
Vortioxetine	2 (6.7%)	0 (0)	
NDRIs	3 (10%)	3 (9.4)	
NaSSa (mirtazapine)	7 (23.3%)	5 (15.6)	
Lithium	1 (3.3%)	1 (3.1)	
Antipsychotics	8 (26.7%)	17 (53.1)	
TCAs	1 (3.3%)	2 (3.1)	

Abbreviations: ASD = autism spectrum disorder; ADHD = attention-deficit/hyperactivity disorder; BD = bipolar disorder; NaSSa = noradrenergic and specific serotonergic antidepressant; NDRIs = norepinephrine–dopamine reuptake inhibitor; PD = personality disorder; SD = standard deviation; SNRIs = serotonin–norepinephrine reuptake inhibitors; SSRIs = selective serotonin reuptake inhibitors; TCAs = tricyclic antidepressants; TD = temporary disability.

## Data Availability

The datasets generated and analyzed during the current study are available from the corresponding author on reasonable request.
